# Continued adherence to community-based health insurance scheme in two districts of northeast Ethiopia: application of accelerated failure time shared frailty models

**DOI:** 10.1186/s12939-022-01620-9

**Published:** 2022-02-05

**Authors:** Mohammed Hussien, Muluken Azage, Negalign Berhanu Bayou

**Affiliations:** 1 Department of Health Systems Management and Health Economics, School of Public Health, College of Medicine and Health Sciences, Bahir Dar University, Bahir Dar, Ethiopia; 2 Department of Environmental Health, School of Public Health, College of Medicine and Health Sciences, Bahir Dar University, Bahir Dar, Ethiopia; 3 Department of Health Policy and Management, Faculty of Public Health, Institute of Health, Jimma University, Jimma, Ethiopia

**Keywords:** Community-based health insurance, Membership adherence, Ethiopia

## Abstract

**Background:**

The sustainability of a voluntary community-based health insurance scheme depends to a greater extent on its ability to retain members. In low- and middle-income countries, high rate of member dropout has been a great concern for such schemes. Although several studies have investigated the factors influencing dropout decisions, none of these looked into how long and why members adhere to the scheme. The purpose of this study was to determine the factors affecting time to drop out while accounting for the influence of cluster-level variables.

**Methods:**

A community-based cross-sectional study was conducted among 1232 rural households who have ever been enrolled in two community-based health insurance schemes. Data were collected using an interviewer-administered questionnaire via a mobile data collection platform. The Kaplan–Meier estimates were used to compare the time to drop out among subgroups. To identify predictors of time to drop out, a multivariable analysis was done using the accelerated failure time shared frailty models. The degree of association was assessed using the acceleration factor (δ) and statistical significance was determined at 95% confidence interval.

**Results:**

Results of the multivariable analysis revealed that marital status of the respondents (δ = 1.610; 95% CI: 1.216, 2.130), household size (δ = 1.168; 95% CI: 1.013, 1.346), presence of chronic illness (δ = 1.424; 95% CI: 1.165, 1.740), hospitalization history (δ = 1.306; 95% CI: 1.118, 1.527), higher perceived quality of care (δ = 1.322; 95% CI: 1.100, 1.587), perceived risk protection (δ = 1.218; 95% CI: 1.027, 1.444), and higher trust in the scheme (δ = 1.731; 95% CI: 1.428, 2.098) were significant predictors of time to drop out. Contrary to the literature, wealth status did not show a significant correlation with the time to drop out.

**Conclusions:**

The fact that larger households and those with chronic illness remained longer in the scheme is suggestive of adverse selection. It is needed to reconsider the premium level in line with household size to attract small size households. Resolving problems related to the quality of health care can be a cross-cutting area of ​​intervention to retain members by building trust in the scheme and enhancing the risk protection ability of the schemes.

## Introduction

Universal health coverage requires that everyone in a country has adequate access to essential health care without financial difficulty, regardless of living standards [1]. Moving to universal health coverage requires a strong health system with stable financing mechanisms [2]. A growing number of low and middle-income countries, including Ethiopia, are implementing voluntary community-based health insurance (CBHI) schemes as a risk-pooling mechanism for rural communities and informal sector workers [3, 4]. In most CBHI schemes, the issue of financial sustainability becomes a critical challenge to achieve the goal of universal health coverage [4, 5].

The sustainability of a voluntary CBHI scheme depends to a greater extent on its capacity to retain members. While initial uptake is important, continued membership adherence is vital to establish a stable insurance scheme that can generate adequate funding for health care [6]. The key indicators for measuring membership that determine the sustainability of voluntary insurance schemes are growth rate, coverage ratio, and renewal rate [6–9]. Growth rate measures how fast the number of insurance members is increasing or decreasing over time, while coverage ratio measures the proportion of the target population participating in the scheme [6, 7]. The renewal rate measures the proportion of insured that stay enrolled in the scheme after their coverage term expires. It is an indicator of scheme performance in retaining its members [7, 9]. High renewal rates indicate that members value services, find the premiums affordable, and are satisfied with the scheme benefits and health services, while low renewals reflect that the target population is not satisfied enough to maintain its membership [6, 7].

An increase in membership growth and renewal rates results in increased revenue, lower marginal cost, and lower health care costs as we can retain relatively healthy members [6, 7, 10]. In a voluntary health insurance scheme, a lower renewal rate leads to adverse selection, that is, healthy members drop out of the scheme while high-risk groups are more inclined to maintain their membership, leading to the existence of small pools with little risk redistribution capabilities [11]. This drives healthcare and administrative costs to higher than anticipated levels, which eventually hamper the financial sustainability of the scheme [10]. In such circumstances, health insurance schemes will fall short of improving access to health care and protecting members from financial hardship [12].

Apart from a few successful cases, CBHI initiatives face the issue of persistently low membership [4], which could be attributed to either an initial low enrollment or high dropout rate [6, 7]. Member dropout has been a great concern for CBHI schemes in low- and middle-income countries. Prior studies that have dealt with this issue reported a higher rate of dropout. For instance, in Bangladesh, a drop-out rate of 62% was reported [13], while in India a dropout rate of 63% was documented in 2013 for three CBHI schemes [14]. In Ghana, the dropout rate ranged between 34.8% in 2012 [15] to 53% in 2016 [16]. Recent work in Uganda estimated that 25.1% of households that had ever enrolled in voluntary CBHI reported dropping out [17]. Although a dropout rate of only 18% was reported one year after the initiation of CBHI in piloted districts of Ethiopia [18], later studies revealed higher figures. Among households that had ever enrolled in the scheme, 31.9% [19] and 37.3% [20] were dropouts. It could be argued that the higher renewal rate during the pilot phase of the scheme was due to a campaign effect following a coordinated effort by the government and non-governmental organizations to enhance its acceptability by the community [18].

It is imperative to generate a comprehensive set of empirical evidence on the factors that influence continued membership adherence to CBHI schemes. Even though several studies have been carried out on the factors influencing dropout decisions [13, 14, 16, 18, 21–26], none of these looked into the ability of the schemes to retain members in the long term, that is, how long members adhere to the scheme and the factors associated with the time to drop out. Moreover, earlier studies have ignored the effect of cluster-level variables on dropout decisions. Therefore, considering the hierarchical nature of the data, this study attempted to identify the factors influencing the time to drop out of a CBHI scheme using a shared frailty survival model.

Ethiopia's current health care financing strategy aims to gradually establish a unified national risk pool system to allow cross-subsidy between high-risk and low-risk areas [27]. The results of this study will be an essential input for policymakers to devise membership retention strategies in an attempt to establish higher-level pools at various levels of administration.

### Design of the CBHI scheme in Ethiopia

The CBHI scheme is run by the government with community involvement in its design, management, and supervision. Enrollment is voluntary and membership needs to be renewed annually with the willingness of the members. To minimize adverse selection, the unit of enrollment is at the household level [28, 29].

At the time of the pilot phase, premiums ranged between $7.31 to $10.45 per year per household across regions. Regions have the freedom to modify the premium based on the local context [28, 29]. For example, in the Amhara National Regional State where this study was conducted, the initial annual premium of $8.36 was later revised to different levels of contributions that vary based on household sizes. In the rural setups, the annual premiums range between $8.54 to $12 [30].

To enhance the affordability of health care, a general subsidy is provided to the scheme by the federal government that constitutes 25% of overall enrollment contributions per year. A targeted subsidy from the regional and district governments is also provided to cover the costs of fee waiver to 10% of the population who are indigent groups. The CBHI benefit package covers all outpatient and inpatient services at the health center and hospital level except for treatment abroad, procedures with large cosmetic values such as tooth implantation, eyeglasses, and plastic surgery; organ transplantation; chronic renal dialysis and treatment for non-generic medicines [28, 29].

## Methods

### Study setting

The study was conducted in the rural parts of two neighboring districts in northeast Ethiopia, Tehulederie and Kalu. Tehulederie is divided into 20 rural and 7 urban *kebeles* (subdistricts) with an estimated total population of 145,625 of which 87.5% are residing in the rural area. In the district, there are five health centers and one primary hospital. Tehulederie was one of the 13 districts in Ethiopia, where the CBHI scheme was piloted in 2011. After two years of implementation of the pilot project, enrollment in the scheme reached 91% [28]. However, membership coverage had declined to 60% among 22,678 eligible households as of April 2020 [31].

Based on lessons learned from the evaluation findings of the CBHI pilot project in the country, a decision was made to design and implement a national scale-up of the initiative to 161 districts in July 2013 including Kalu district [28]. Kalu is divided into 36 rural and 4 urban *kebeles* and has nine health centers that provide health care to the surrounding area. It is the most populous district in the zone, which has an estimated total population of 234,624 among which 89.11% are living in the rural part. Among 46,924 eligible households in the district, 61% were covered by CBHI after seven years of implementation in 2020 [31].

### Study design and population

A community-based cross-sectional study was conducted using face-to-face interviews to collect quantitative data among rural households who had ever been enrolled in CBHI before January 2020. The study focused on rural *kebeles* because the scheme in urban *kebeles* had a unique design, and was started recently which makes it too early to evaluate its sustainability. Non-paying scheme members were excluded from the study since such members have no incentive to drop out of the scheme, the inclusion of which could have a confounding effect on the association between economic status and time to drop out. Because CBHI membership was at the household level, data were collected and analyzed at the household level.

### Study variables and measurement

The outcome variable was time to drop out of a CBHI membership starting from the point at which the households joined the scheme and was measured in years. The event of interest was dropping out of CBHI membership, hence households that dropped out were coded as “1” and otherwise “0”.

The independent variables for this study were selected based on the findings of a systematic review. We did a systematic review of the factors that influence CBHI membership renewal in low- and middle-income countries before starting this study [32]. Search strategies for electronic databases were developed in line with the PRISMA guidelines [33] for systematic reviews and a search was conducted from Dec. 2019 – Feb. 2020, using PubMed, Scopus, and Hinari electronic databases. The reference lists of relevant systematic reviews and the studies included in the review were checked for additional papers. The details are available at https://doi.org/10.2147/CEOR.S306855.

The results of the systematic review revealed that age [22, 25, 34–36], gender [16, 21, 36], level of education [13, 14, 22–24], and marital status [21, 22] of the household head; household size [22, 35]; wealth status [13, 14, 22, 26, 34]; place of residence [23, 24, 26]; distance to the nearest health facility [13, 25, 34]; participation in a safety net program [18]; self-rated health status [21, 22]; recent episodes of illness [18, 25]; presence of chronic illness in the household [18, 24, 26]; history of hospitalization [14]; use of health care under the scheme [18, 24, 36]; frequency of health facility visit [13, 36]; benefit claims experience [13, 14]; perceived quality of health care [23–25]; value towards solidarity [25]; perceived risk protection [21] and trust in the scheme [25, 37] were important factors influencing the renewal decision of CBHI members.

With a few exceptions, we included all of the above variables in this study. Because the whole population of interest lives in a rural setting, place of residence was not taken into account in this study. Distance to a health facility is an important variable linked to renewal decisions. However, it was omitted in our case since it was considered as a *kebele* level variable that might be handled by the frailty analysis as unobserved characteristics (assuming people in the same *kebele* are located nearly at the same distance to the health facility). We did not include recent illness episodes as a predictor variable since we believed it could not impact previous dropout decisions.

During the pretest, we attempted to gather data on the frequency of health facility visits, usage of health care under the scheme, and benefit claims experience, but we were unable to get meaningful data due to recall bias. Furthermore, we sought to obtain the yearly average health facility visits and benefit claims from the membership registration files of the schemes. We also failed to obtain the necessary data in this area owing to incomplete recording. As a result, we decided to omit these factors from the study.

Wealth index was generated using the principal component analysis method. The scores for 15 types of assets and utilities were translated into latent factors and a wealth index was calculated based on the first factor that explained most of the variation. Based on the index the study households were categorized into wealth tertile – lower, medium, and higher wealth tertile. Participation in a safety net program refers to being covered by a program catering to food-insecure households.

Self-rated health status was measured based on a household head’s subjective assessment of the health status of the household and was rated as “excellent, very good, good, fair, or poor”. However, for analysis purposes, it was recategorized into fair, good, and very-good, by merging the two extreme response categories with few frequencies to the next categories. The presence of chronic illness refers to one or more members of the household having a known chronic illness that require ongoing medical attention and being informed by a health care provider. History of hospitalization was measured by asking the respondents whether or not any member of the household had received inpatient service after joining the scheme.

Value towards solidarity, perceived risk protection, and trust in the scheme are composite variables that were measured on a Likert scale using a 5—point response format with 1 = strongly disagree, 2 = disagree, 3 = neutral, 4 = agree, and 5 = strongly agree by asking respondents to rate the extent to which they agreed on a set of items designed for each variable. To measure value towards solidarity, a three-item tool was adapted from a previous study conducted in Senegal [38], while a four-item trust measurement scale was adapted based on a previous tool validated and used in Cambodia [37]. Perceived risk protection is the perception of insurance members towards the ability of the CBHI scheme to protect subscribers from financial risks. It was measured using three items, which include the scheme being able to protect members from out-of-pocket expenditure, from selling their important assets, or borrowing money at the time of receiving health care. An overall index was calculated from a set of items using factor analysis, and a three-level categorical variable labeled as “low, medium, and high” was created for each of the three variables.

Perceived quality of health care was measured on a Likert scale using a 5—point response format with 1 = strongly disagree to 5 = strongly agree by asking respondents to rate the extent to which they agreed on a set of nine experience questions regarding the health services they received from the nearby health centers contracted by the CBHI scheme. The scores for the nine items were translated into three dimensions, and an overall health care quality index was created based on the first dimension that explained most of the variation. Finally, the health care quality index was categorized into low, medium, and high.

### Sample size and sampling procedure

The sample size was calculated using MedCalc sample size calculator software version 20 via the log-rank test which compares survival rates of two independent groups. Due to the absence of previous studies on CBHI that could be used as an input for sample size calculation in survival analysis, a survival rate of 0.50, which yields an adequate sample size was assumed for the unexposed group. The survival rate in the exposed group was set to be 0.60 to achieve a power that could detect at least a 10% difference in survival rates between the two groups. This yields an effect size of 0.2037, which is the smallest recommended effect size for calculating an adequate sample size [39]. Hence, it was assumed that there is a binary variable that divides the sample population into two equal groups with survival rates of 0.50 in the unexposed group and 0.60 in the exposed group, 80% statistical power, and 95% confidence level [39]. Based on these assumptions, a sample size of 762 was calculated. Considering a design effect of 1.5 attributed to the use of a multi-stage sampling and a potential non-response rate of 10%, the effective sample size was estimated to be 1257 households.

The study participants were recruited using a three-level multistage sampling method. First, 12 clusters of *kebeles* organized under a health center catchment area were selected. Then, 14 rural *kebeles* were drawn randomly using a lottery method proportional to the number of *kebeles* under each cluster. Accordingly, five *kebeles* from Tehulederie and nine from Kalu were included. A list of households who have ever been enrolled in the CBHI scheme was obtained from the membership registration books of each *kebele* and these lists were used as the sampling frame. Then, 1257 households were drawn randomly using a random number generator software from the selected *kebeles* proportional to the total number of households ever enrolled in the scheme.

### Data collection process

Household-level data were collected from 04 February to 21 March 2021 using a structured interviewer-administered questionnaire by trained data collectors. Information related to membership duration and status of the households was obtained from the membership registration book at each *kebele* (health post), which provides data related to the annual renewal status since the initiation of the scheme. Ten years of follow-up data were obtained starting from the initiation of the CBHI scheme in 2011 to the 2021 registration period, after which renewal data were not available. Information related to socio-demographic characteristics of the household, health status, health care utilization, value towards solidarity, perceived risk protection, trust in the scheme, and perception of respondents towards health care quality were collected at the household level through face-to-face interviews. The heads of the households were interviewed at their home or workplace using the local language, Amharic. The data collectors were guided by health extension workers to track the sampled households. Health extension workers provide home-based health services in rural *kebeles* and are well aware of the addresses of each household. A mobile data collection platform, Open Data Kit (ODK) was applied to the household survey. The data collectors submitted the completed forms to the ODK aggregator (Kobo) server daily, which helped us to review the daily submissions and facilitate the supervision process.

Before the data collection, the questionnaire was pre-tested on a sample of 84 randomly selected participants in one *kebele*. As part of the pre-test, a cognitive interview was conducted on selected items using the verbal probe technique among eight respondents to determine whether or not items and response categories were understood and interpreted by the potential respondents as intended. Accordingly, the wording of some items and response options were modified and some items were removed.

### Data processing and analysis

The data were analyzed using Stata version 17.0. Exploratory factor analysis was performed to assess the validity of the questionnaires designed to measure value towards solidarity, perceived risk protection, trust in the scheme, and perceived quality of health care. The Bartlett’s test of Sphericity and the Kaiser-Mayer-Olkin’s (KMO) measure of sampling adequacy tests were performed to assess the appropriateness of the data for factor analysis. Items with insignificant loadings (loading below 0.40) and items with a cross-loading were removed from the analysis. The Eigenvalue greater than one decision rule was used to determine the appropriate number of factors to be extracted. The reliability of measurement scales was estimated by measuring the internal consistency of each of the dimensions using Cronbach’s alpha, with an acceptable alpha value of 0.60 or higher [40].

The total membership-years of follow-up with an average follow-up time and the annual dropout rate were computed. The time to drop out of a CBHI membership was described using the Kaplan–Meier estimate. To investigate the effect of variables on the time to drop out of CBHI members, a univariate analysis was performed by fitting separate models for each variable before proceeding to the multivariate analysis. Variables that were significant in the univariate analysis at a p-value of less than 0.20 [41] were included in the multivariable analysis. A multivariable analysis was done using the accelerated failure time shared frailty models to identify the predictors of time to drop out.

The classical Cox proportional hazard model, which is commonly used to analyze survival data, assumes that the ratio of the hazards between any two individuals is constant over time. However, in many applications, the study population cannot be considered homogeneous. In this study, the time to drop out of a CBHI membership is assumed to be different between clusters (*kebeles*) due to variations in the performance of the *kebele* health insurance committee which is mainly responsible for retaining scheme members. The intra-cluster correlation is assumed to be due to unobservable covariates specific to the cluster. One approach to account for such unobserved heterogeneity is the use of a shared frailty model which introduces a random effect into the model that induces dependence within clusters. In a shared frailty model, individuals in a cluster are assumed to share the same frailty value [42].

Frailty is an unobservable random effect shared by subjects within a cluster. It acts multiplicatively on the hazard function assumed to follow some distribution. When a shared frailty term with a Weibull distribution is assumed, the hazard function at time *t* for the *j*^*th*^ individual, *j* = 1, 2,..., *n*_*i*_, in the *i*^*th*^ group, i = 1, 2,..., *g*, is given by:

1 $${h}_{ij}(t)={z}_i\mathit{\exp}\left({\beta}^{\prime }{x}_{ij}\right)\rho {t}^{\rho -1},$$

where *x*_*ij*_ is a vector of explanatory variables for the *j*^*th*^ individual in the *i*^*th*^ group, *β* is the vector of regression coefficients, ρt^ρ−1^ is the baseline hazard function, ρ a shape parameter and the *z*_*i*_ are frailty effects that are common for all *n*_*i*_ individuals within the *i*^*th*^ group [43].

The hazard function can also be written in the form:

2 $${h}_{ij}(t)=\mathit{\exp}\left(\beta^{\prime }{x}_{ij}+{u}_i\right)\rho {t}^{\rho -1},$$

where u_i_ = log(z_i_).

The corresponding survivor function for a Weibull model that incorporates a shared frailty component is:

3 $${S}_{ij}(t)=\mathit{\exp}\left\{-\mathit{\exp}\left({\beta}^{\prime }{x}_{ij+}{u}_i\right){t}^p\right\},$$

The frailty is generally assumed to follow a gamma or inverse-Gaussian distribution with a mean equal to 1, and variance θ which is estimated from the data. The estimate for the variance parameter θ in a shared frailty model can be thought of as a measure of the degree of correlation, where θ > 0 indicates the presence of heterogeneity. Large values of θ reflect a greater degree of heterogeneity among clusters and a stronger association within clusters [43].

An accelerated failure time (AFT) model is a parametric model that provides a useful alternative to the commonly used proportional hazards models in survival analysis owing to its ease of interpretation. In addition, the regression parameters in AFT models are robust towards omitted covariates unlike that of the proportional hazards models [44]. The AFT model is a general model for survival data in which explanatory variables measured on an individual are considered to act multiplicatively on the timescale. It allows researchers to measure the direct effect of predictor variables on survival time. In contrast to the proportional hazards model, the AFT model can best be interpreted in terms of the survival function [45]. The AFT model is defined by the relationship:

4 $${S}_1(t)={S}_0\left(t/\delta \right), for\ t>0,$$

where δ is a constant called the acceleration factor, which tells the researcher how the change in the value of the covariate changes the time scale relative to the baseline time scale. The acceleration factor is the ratio of the survival time corresponding to any fixed value of S(t). In the regression framework, the acceleration factor δ can be parameterized as exp (α), where α is the parameter to be estimated from the data. With this parameterization, the general form of the survivor function for the *i*^*th*^ individual in an AFT model is:

5 $${S}_i(t)={S}_0\left\{t/\mathit{\exp}\left(\alpha^{\prime }{x}_i\right)\right\},$$

In this version of the model, *exp(α′x*_*i*_*)* is the acceleration factor for the *i*^*th*^ individual.

The general parametric AFT model that incorporates a shared frailty component is of the form:

6 $${S}_{ij}(t)={S}_0\left\{t/\mathit{\exp}\left({\eta}_{ij}\right)\right\},$$

where *η*_*ij* =_*α′x*_*ij*_ + *ui,* and *exp* (*ηij*) is the acceleration factor for the *j*^*th*^ individual in the *i*^*th*^ group. This model can be expressed in log-linear form as:

7 $$\mathit{\log}{T}_{ij}=\mu +{\alpha}_1{x}_{1 ij}+{\alpha}_2{x}_{2 ij}+\dots +{\alpha}_p{x}_{pij}+{u}_i+\gamma {\epsilon}_{ij},$$

where *T*_*ij*_ is the random variable associated with the survival time of the *j*^*th*^ individual in the *i*^*th*^ group, *μ* and *γ* are intercept and scale parameters respectively and *u*_*i*_*’s* are the cluster-specific random effects. The quantity *ϵ*_*ij*_ is a random variable used to model the deviation of the values of *logT*_*ij*_ from the linear part of the model, and *ϵ*_*ij*_ is assumed to have a particular parametric distribution [46]. In this formulation of the model, the *α*-parameters reflect the effect that each explanatory variable has on the survival times; positive values suggest that the survival time increases with increasing values of the explanatory variable and vice versa.

The common baseline distributions of the AFT models include exponential AFT, Weibull AFT, log-logistic AFT, and log-normal AFT distributions. Akaike’s Information Criterion (AIC) and Bayesian Information Criterion (BIC) were used for model comparison. The overall fit of the final AFT model was checked by using the Cox-Snell residuals plot. Finally, the variance of the random effect (θ), Kendall’s Tau (τ), the regression coefficients, and the acceleration factor (δ) with 95% confidence interval were estimated.

## Results

### Baseline characteristics of the study population

A total of 1232 ever enrolled CBHI members participated in this study with a response rate of 98%. The average age of the study participants was 49.45 years (SD = 12.25), with slightly more than half (50.65%) being between the ages of 45 and 64, and 13.72% being 65 and older. Among the total household heads, 1064 (86.36%) were males, and 1132 (91.88%) were currently married. More than three-quarters of the study participants (78.90%) did not attend formal education, and a larger proportion of them (60.88%) had a household size of five and above.

As for the health status, nearly one-fourth of the households (23.70%) had one or more individuals with a known chronic illness, while 490 (39.77%) of the households had received inpatient service under the CBHI scheme. Moreover, a smaller proportion of the households (17.69%) rated their household health status as fair, while 566 (45.94%) and 448 (36.36%) of them rated it as good and very good respectively (Table 1).

**Table 1:** Characteristics of households ever enrolled in a CBHI scheme in two districts of Northeast Ethiopia

Variables	Categories	Renewals /Censored, N(%)	Dropouts /Events, N(%)	Total N(%)
Age in years	25 – 44	319 (36.54)	120 (33.43)	439 (35.63)
	45 – 64	430 (49.26)	194 (54.04)	624 (50.65)
	65 +	124 (14.20)	45 (12.53)	169 (13.72)
Gender	Male	772 (88.43)	292 (81.34)	1,064 (86.36)
	Female	101 (11.57)	67 (18.66)	168 (13.64)
Marital status	Divorced or widowed	48 (5.50)	52 (14.48)	100 (8.12)
	Married	825 (94.50)	307 (85.52)	1,132 (91.88)
Educational status	No formal education	689 (78.92)	283 (78.83)	972 (78.90)
	Attend formal education	184 (21.08)	76 (21.17)	260 (21.10)
Household size	< Five	323 (37.00)	159 (44.29)	482 (39.12)
	≥ Five	550 (63.00)	200 (55.71)	750 (60.88)
Wealth tertile	Lowest	271 (31.04)	139 (38.72)	410 (33.28)
	Medium	294 (33.68)	117 (32.59)	411 (33.36)
	Highest	308 (35.28)	103 (28.69)	411 (33.36)
Participation in a Safety net program	No	743 (85.11)	318 (88.58)	1,061 (86.12)
	Yes	130 (14.89)	41 (11.42)	171 (13.88)
Self-rated health	Fair	159 (18.21)	59 (16.43)	218 (17.69)
	Good	408 (46.74)	158 (44.01)	566 (45.94)
	Very good	306 (35.05)	142 (39.55)	448 (36.36)
Chronic illness	No	642 (73.54)	298 (83.00)	940 (76.30)
	Yes	231 (26.46)	61 (17.00)	292 (23.70)
Hospitalization	No	488 (55.90)	254 (70.75)	742 (60.23)
	Yes	385 (44.10)	105 (29.25)	490 (39.77)
Perceived quality of health care	Low	283 (32.42)	133 (37.05)	416 (33.77)
	Medium	292 (33.45)	115 (32.03)	407 (33.04)
	Haigh	298 (34.14)	111 (30.92)	409 (33.20)
Value towards solidarity	Low	283 (32.42)	137 (38.16)	420 (34.09)
	Medium	382 (43.76)	140 (39.00)	522 (42.37)
	Haigh	208 (23.82)	82 (22.84)	290 (23.54)
Perceived risk protection	Low	256 (29.32)	157 (43.73)	413 (33.52)
	Medium	201 (23.02)	83 (23.12)	284 (23.05)
	Haigh	416 (47.65)	119 (33.15)	535 (43.43)
Trust in scheme	Low	245 (28.06)	159 (44.29)	404 (32.79)
	Medium	287 (32.88)	124 (34.54)	411 (33.36)
	Haigh	341 (39.06)	76 (21.17)	417 (33.85)
District	Tehulederie	306 (64.69)	167 (35.31)	473 (38.39)
	Kalu	567 (74.70)	192 (25.30)	759 (61.61)
	**Total**	**873 (70.86)**	**359 (29.14)**	**1,232 (100)**

### Time to drop out of CBHI scheme among the study population

Among the 1232 study participants, 29.14% (95% CI: 26.61%, 31.77%) had dropped out of CBHI following their initial enrollment in the scheme with an incidence rate of 5.27 per 100 person-year (95% CI: 4.75, 5.84). A quarter of respondents (25.30%) from Kallu and more than one-third (35.31%) from Tehulederie had dropped out of the scheme. The total follow-up period was 6816 person-years of observation, with an average follow-up time of 5.53 years (95% CI: 5.38, 5.68).

**Fig. 1:**
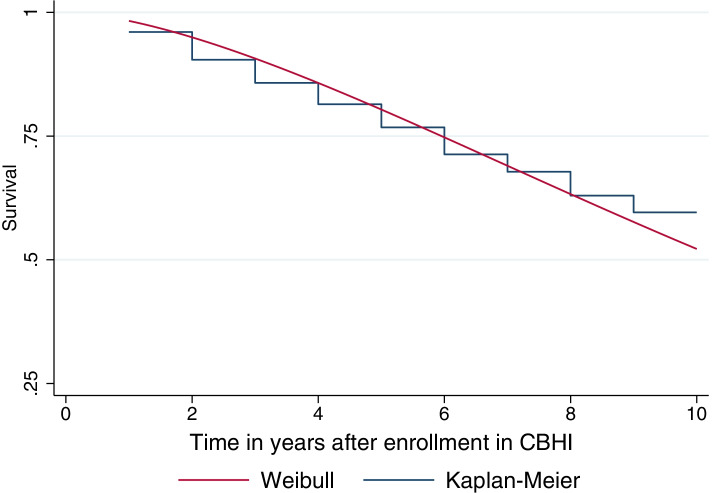
Kaplan–Meier curve (stairstep line) and Weibull survival plot (solid line) for the time to drop out of CBHI membership after initial enrollment

Kaplan–Meier estimates were used to plot the survival function for the time to drop out of CBHI. As shown in Fig. 1, the probability of remaining in the scheme beyond 10 years was 59.6%. The median survival time was not reached, because more than 50% of the study participants remained in the scheme beyond the follow-up time.

To describe how the time to drop out of CBHI was distributed by different variables, Kaplan–Meier curves were plotted for selected variables that were significant at p-value < 0.05 based on the log-rank test (Fig. 3). For the first 10 months after initial enrollment, the survival curves for respondents who were male, and married were consistently above those who were female and divorced or widowed. There exists also a clear difference in the survival curve of households having a chronic illness and those who had received inpatient service under CBHI compared to their reference groups, both of which prolong the time to drop out. Similarly, the survival curves for respondents who had a high-level perception on the quality of health care and the risk protection ability of the scheme, and who had high-level trust in the scheme were above their counterparts.

### Model selection

Multivariable AFT models of exponential, Weibull, lognormal, and loglogistic baseline hazard functions were fitted by considering both the gamma and inverse-Gaussian frailty distributions. The Weibull AFT inverse-Gaussian shared frailty model, which had a minimum AIC and BIC values was the preferred model to analyze the data. The AIC and BIC values of the different parametric AFT models with gamma and inverse-Gaussian shared frailty distributions are displayed in Table 2.

**Table 2:** Comparison of the different parametric AFT shared frailty models based on the AIC and BIC values

Baseline distribution	Frailty distribution	AIC	BIC	Variance (θ)	p-value of LR test of θ = 0
Exponential	Gamma	1858.14	1945.11	0.080	< 0.001
	Inverse-Gaussian	1857.50	1944.48	0.084	< 0.001
Weibull	Gamma	1779.42	1871.52	0.117	< 0.001
	Inverse-Gaussian	1779.13	1871.23	0.126	< 0.001
Lognormal	Gamma	1787.85	1879.94	0.101	< 0.001
	Inverse-Gaussian	1787.45	1879.55	0.106	< 0.001
Loglogistic	Gamma	1785.84	1877.93	0.107	< 0.001
	Inverse-Gaussian	1785.37	1877.46	0.112	< 0.001

The four parametric AFT baseline distributions with Gamma and inverse-Gaussian frailty distributions were fitted by using *kebele* as a frailty component. The frailty effect was statistically significant for all the parametric survival AFT models, both in the null and full models. The frailty in the final model is assumed to follow an inverse-Gaussian distribution with a mean of 1 and a variance equal to theta (θ). The variance of the random effect (θ = 0) would mean that the frailty component does not contribute to the model. The estimated variability (heterogeneity) in the population of clusters (*kebeles*) using the Weibull inverse-Gaussian shared frailty model was 0.126, which means that 12.6% of the variation in the time to drop out was accounted for unobservable cluster-level factors. The likelihood ratio test that assumes a variance of frailty θ = 0 resulted in a highly statistically significant p-value of < 0.001, indicating that the frailty component has a significant contribution to the model and that there is an intra-cluster correlation. The estimated value of the variance of the frailty in the null model was 0.161. The associated Kendall’s tau (τ), which measures the intra-cluster dependence was estimated to be 0.075 and 0.059 for the null and full models respectively. The value of the shape parameter in the Weibull inverse-Gaussian shared frailty model was greater than unity (*ρ* = 1.573, its p-value < 0.001), which indicates that the hazard increases to a maximum point and then decreases over time, resulting in a unimodal hazard function which is due to the frailty effect [43].

**Fig. 2:**
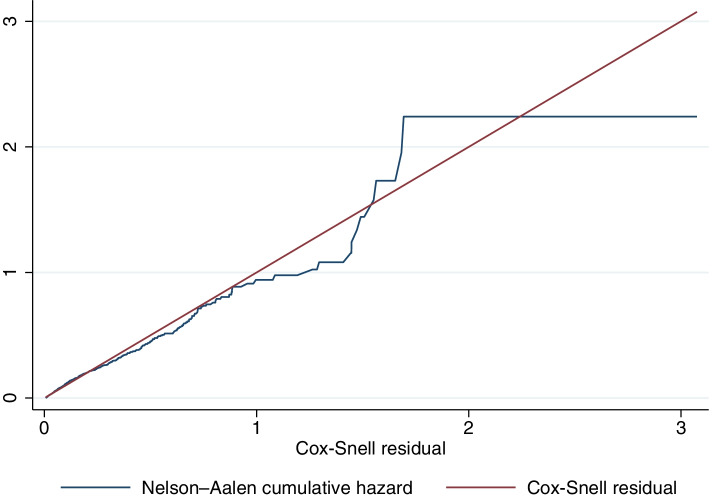
Cumulative hazard plot of Cox-Snell residual for the Weibull AFT model

The goodness of fit of the final model was checked using the Cox–Snell residuals plot. The Cox-Snell residuals plot of the Weibull AFT inverse-Gaussian frailty model was closer to the 45-degree straight line, with a slight deviation in the right tail, suggesting that the model well-fitted the time to drop out data (Fig. 2). Some variability is expected at the 45-degree straight line, especially in the right tail, although we have a well-fitting model. This deviation was due to reduced effective sample size caused by earlier dropouts [47].

### Analytical results of the Weibull AFT inverse-Gaussian shared frailty model

Based on the values of the AIC and BIC, and variance of the frailty effect, the Weibull inverse-Gaussian shared frailty model was selected as the preferred parametric survival model to analyze the data. Variables with a p-value of < 0.20 in the univariate analysis of the Weibull inverse-Gaussian shared frailty model were included in the multivariable analysis.

If the hierarchical nature of the data was not taken into account while doing the univariate and multivariable analysis of the survival models, it makes sense to perform a separate analysis for each district. However, because the frailty survival model takes cluster (sub-district) level characteristics into account, we analyze it by combining data from the two districts.

**Fig. 3:**
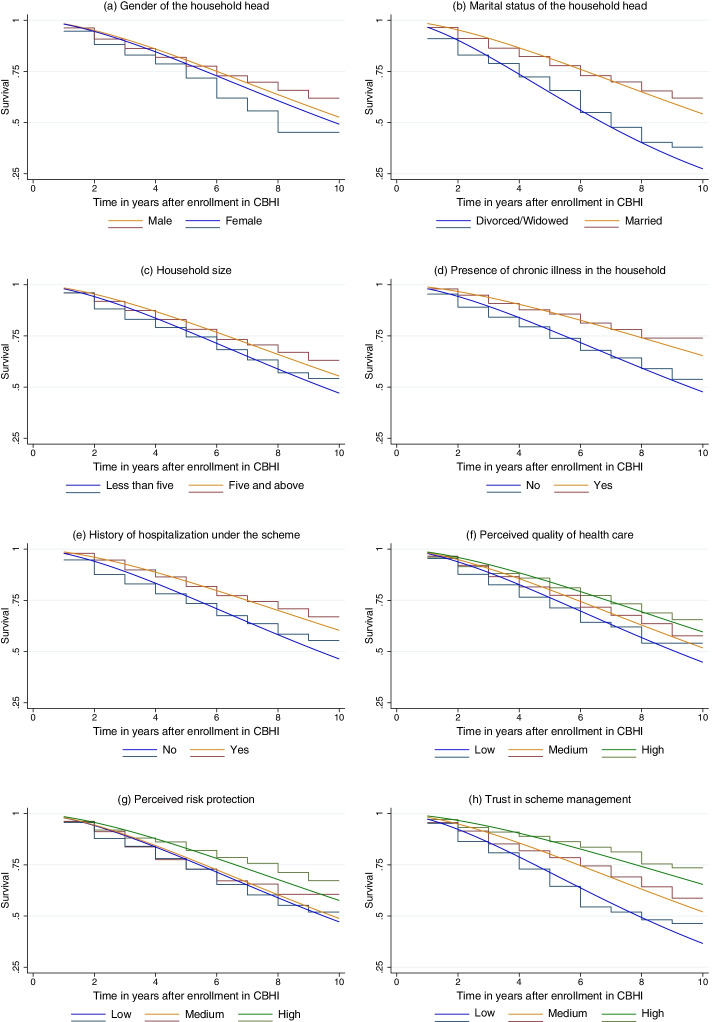
Kaplan–Meier curves (stairstep lines) and Weibull survival plots (solid lines) for selected variables associated with the time to drop out of CBHI

In the univariate analysis, age, gender, and marital status of the household head; household size; self-rated health status; the presence of chronic illness in the household; history of hospitalization under the scheme; perceived quality of health care; perceived risk protection; and trust in the scheme were significantly associated with time to drop out at p-value < 0.05, while the educational status of the household head, wealth tertile, participation in a safety net program and value towards solidarity were not statistically significant at p-value < 0.20. After fitting the multivariable inverse-Gaussian shared frailty model, the Weibull distributions of selected variables were plotted over the respective Kaplan–Meier curves to show how well the estimated Weibull survival plots fitted the data (Fig. 3).

After adjusting for other independent variables, and keeping households in the same cluster, all variables included in the multivariable analysis were significantly associated with the time to drop out of the CBHI scheme at 95% confidence level, except age, gender, and self-rated health status (Table 3). Accordingly, the time to drop out for married household heads was increased by a factor of 1.610 compared to their counterparts of divorced or widowed household heads (δ = 1.610; 95% CI: 1.216, 2.130). Scheme members having larger household sizes adhere to the scheme longer compared to those having smaller household sizes with an acceleration factor of 1.168 (δ = 1.168; 95% CI: 1.013, 1.346).

**Table 3:** Multivariable analysis using the Weibull inverse-Gaussian shared frailty model on predictors of time to drop out

Variables	Categories		Coef	S. E		δ	*p*-value	95% CI for δ
Intercept			1.356	0.205		3.879	0.000	(2.598, 5.793)
Age in years	25–44		ref					
	45–64		-0.014	0.076		0.986	0.850	(0.849, 1.145)
	65 +		0.172	0.119		1.188	0.148	(0.941, 1.501)
Gender	Male		ref					
	Female		-0.063	0.124		0.939	0.611	(0.735, 1.198)
Marital status	Divorced/widowed		ref					
	Married		0.476	0.143		1.610	0.001	(1.216, 2.130)
Household size	Smaller (< 5)		ref					
	Larger (≥ 5)		0.155	0.072		1.168	0.032	(1.013, 1.346)
Self-rated health	Fair		ref					
	Good		0.037	0.108		1.037	0.736	(0.839, 1.282)
	Very good		-0.081	0.113		0.922	0.471	(0.739, 1.150)
Chronic illness	No		ref					
	Yes		0.353	0.102		1.424	0.001	(1.165, 1.740)
Hospitalization	No		ref					
	Yes		0.267	0.080		1.306	0.001	(1.118, 1.527)
Perceived quality of health care	Low		ref					
	Medium		0.127	0.085		1.135	0.135	(0.961, 1.340)
	Haigh		0.279	0.093		1.322	0.003	(1.100, 1.587)
Perceived risk protection	Low		ref					
	Medium		0.030	0.094		1.031	0.748	(0.857, 1.240)
	Haigh		0.197	0.087		1.218	0.023	(1.027, 1.444)
Trust in scheme	Low		ref					
	Medium		0.274	0.088		1.315	0.002	(1.107, 1.563)
	Haigh		0.549	0.098		1.731	0.000	(1.428, 2.098)
		ln (ρ) = 0.453 (p < 0.001)			γ = 0.636 (S.E = 0.028)			
		ρ = 1.573 (S.E = 0.069)			τ = 0.059			
		θ = 0.126 (S.E = 0.066)			AIC = 1779.13, BIC = 1871.23			

*CI* Confidence Interval, *coef* regression coefficient, *S.E* Standard error; *δ* Acceleration Factor, *ρ* Shape parameter, *γ* Scale parameter, *γ* = 1/ρ; θ – Variance of the random effect, *τ* Kendall’s tau, τ = θ/θ + 2, where τ = ϵ (0, 1), *ref* reference category; *AIC* Akaike’s Information Criterion, *BIC* Bayesian information criterion

The time to drop out for scheme members who had a known chronic illness increased significantly by an acceleration factor of 1.424 (δ = 1.424; 95% CI: 1.165, 1.740). This shows that years of membership adherence for households who had a chronic illness in their household was extended by 42.4% compared to those without a chronic illness. Similarly, the time to drop out for households who had received inpatient service under the scheme was estimated to be 1.306 times that of households who did not receive inpatient service (δ = 1.306; 95% CI: 1.118, 1.527).

The time to drop out for scheme members who rated the quality of health care as high was estimated to be 1.322 times that of households who rated it as low (δ = 1.322; 95% CI: 1.100, 1.587). The time to drop out for scheme members who had a high-level perception on the risk protection ability of the scheme was estimated to be 1.218 times that of households who had low-level perception (δ = 1.218; 95% CI: 1.027, 1.444). Likewise, the time to drop out for scheme members who had medium and high-level trust in the scheme is extended by 31.5% and 73.1%, respectively compared to those who had low-level trust in the scheme (δ = 1.315; 95% CI: 1.107, 1.563) and (δ = 1.731; 95% CI: 1.428, 2.098), indicating higher adherence to the scheme.

## Discussions

Although there are a handful of studies that investigated the factors that influence membership renewal (dropout), none of these consider the time to drop out among scheme members. Most of these studies also measure membership dropout rates after a few years of scheme implementation. In contrast to most previous studies, our study attempted to elaborate on the factors that are associated with continued adherence (time to drop out) among CBHI members with a 10-year follow-up period. Moreover, this study considered the effect of unobserved cluster-level variables that determine continued membership adherence.

After adjusting for other independent variables, and keeping households in the same cluster, results of the multivariable regression analysis revealed a number of factors associated with the time to drop out of CBHI. Accordingly, married household heads adhere to the scheme longer compared to their counterparts of divorced or widowed household heads. This finding is consistent with a study in Ghana, where married respondents were more likely to renew their health insurance [21]. This could be because married heads of households might have a higher ability to pay for health insurance since marital status has a positive correlation with economic status [48]. Contrary to this, another study in Ghana reported that married migrant head porters were significantly less likely to renew their membership than single migrant head porters [22]. The difference might be attributed to a difference in the study population and scheme design. In the latter study, the study participants were female migrant head porters who were enrolled in the scheme free of charge.

This study found that household size had a positive effect on prolonging the time to drop out of CBHI. The larger the household, the higher the probability of maintaining membership in the scheme. It seems that household size had a localized effect on membership renewal. Different studies reported conflicting evidence on the association between household size and renewal decisions. One study in Ethiopia found that larger households were more likely than relatively smaller households to extend their policy [19]. This finding is also corroborated by a systematic review which showed that household size was a facilitator of renewal decisions [49]. This might be because larger household sizes are prone to higher out-of-pocket health care expenditures [50]. Risk-averse households with larger family sizes might prefer to maintain insurance membership to avoid the risk of catastrophic expenditures.

Contradiction to our finding, other studies conducted elsewhere reported a negative correlation between household size and renewal decision [17, 22, 35]. It could be argued that the subscription fees in the schemes investigated by the later studies were levied according to the size of the household, which resulted in a higher premium for larger households. This in turn might be a barrier to maintaining their membership. That was not the case in the Ethiopian context until the time of this study, where all households with a household size of five or less contribute the same premium ($8.8800 per year) with an addition of only $1.2686 and $2.5371 for the next two levels of contributions for January 2021 renewal period. Although enrollment was set at the household level to limit adverse selection [18], it appears that the premium design in the context of this study creates some other form of adverse selection by retaining relatively larger households that are at higher risk of health care expenditure. Another explanation is the possibility of partial enrollment (enrolling only at-risk individuals like children, elders, and those with illness experiences) to avoid additional payment for a family size larger than five [51]. Further research is recommended to explore this problem, preferably with a qualitative approach. This finding provides lessons for policymakers to devise strategies to attract small size households and minimize potential partial enrollment, which in part can address the problem of adverse selection.

The households’ chronic illness experiences also influence the decision to remain in the scheme. The membership duration for those who had a family member with chronic illness in the household was extended significantly compared to those without chronic illness. This finding complements what has already been found in the literature on the link between the presence of chronic illness in the family and renewal decisions [18, 24, 26]. The result mainly indicates the possible existence of adverse selection, which is a common event in schemes where membership is voluntary and contributions are independent of individual health risks [52]. This phenomenon has two implications that are worth discussing here. From the equity point of view, CBHI is promoting health care access to high-risk subsistence farmers who otherwise would be forced to catastrophic health expenditure or exposed to an increased risk of severe complications for those who forgo treatment due to inability to cover the cost of health care. The second insight is from the scheme’s performance and sustainability perspective. The result points out that the scheme is unable to retain healthy scheme members which raises concerns about the risk profile of members remaining in the scheme. The insurance pool would be left with high-risk individuals who had higher health care needs, for which the premium is insufficient to cover total claims, which in turn could hamper the financial sustainability of the scheme [53].

This study verified that hospitalization, which is both an illness and service utilization indicator, was positively associated with the time to drop out of CBHI. Receiving inpatient service (hospitalization) under CBHI coverage motivates households to extend their membership. A possible explanation for this finding is that those who have been hospitalized under the coverage of the scheme might have enjoyed the benefit of health insurance more than others. They might have received advanced health care that would have cost them excess money or was beyond their ability to cover its cost. Through their experience, they might have the opportunity to learn and understand the basic principles underlying health insurance. Earlier work in India reported that hospitalization under the scheme was negatively associated with renewal probability [14]. The authors argue that the possible reasons could be the poor quality of health care and the negative claims experience faced by scheme members. Scheme managers need to devise mechanisms to maximize the benefit of CBHI to retain their members.

The perception that health care is of good quality is an important enabler of continued membership adherence. Household heads who rated the quality of health care as high adhere to the scheme longer compared to those who rated it as low. The result confirmed that health care quality is an important consideration in the household’s decision to remain as a scheme member. This is consistent with the existing literature [19, 23–25], although different indicators have been used to measure health care quality. Qualitative studies have also documented different issues linked to the quality of health care as important barriers for membership adherence [15, 54–58]. This finding is not surprising, as the quality of health care is central to the success of any community-based and micro health insurance initiatives aimed at achieving universal health coverage [5, 59, 60]. If the health facilities fail to provide high-quality services, the insured will lose trust in the service provider and the insurance plan and will opt for other care alternatives outside the system [61]. Therefore, members will be unlikely to maintain their subscriptions [49, 62]. This is an important area of intervention that policymakers and other relevant stakeholders should focus on to retain CBHI members and assure the sustainability of the scheme. It might be linked to the respondents’ perception of the ability of CBHI to protect them from financial risks and their trust in the scheme.

In the regression analysis, the household head’s perception on the ability of CBHI to protect its members from financial risks was significantly associated with continued membership adherence. Households who had a high-level perception on the risk protection ability of the scheme had a higher survival probability compared to those with low-level perceptions. One of the primary purposes of universal health coverage targeted efforts, including health insurance initiatives is to abolish the requirements to pay directly at the time of health service utilization [1, 63]. This can be achieved through prepayment and pooling approaches that generate stable and sufficient funds for health, which enable contracted health facilities to provide uninterrupted services [64]. Once members of the scheme paid the premium, they would expect to receive health care without the need to pay at the time of receiving health care. If they are required to pay at the time of health service utilization, they might lose their confidence in the scheme and decide to cancel their policy.

Results from this study also indicated that the household head’s trust in the scheme played an important role in extending the time to drop out among scheme members. Policyholders who had medium and high-level trust in the scheme had longer adherence to the scheme compared to those who had low-level trust. This means that subscribers who believe the scheme does good for the community; the scheme will pay for everything it is supposed to, even really expensive treatments; the scheme staff are completely honest and reliable, and the staff care about their health just as much or more than they do; remain long in the scheme compared to their counterparts. Our finding is in line with results from prior studies which showed that trust in the scheme was a significant enabler of membership adherence [19, 25, 37]. This finding is also supported by findings of meta-analysis and systematic reviews which revealed that trust in insurance schemes was a facilitator of renewal decisions [49].

One fascinating issue in our study is the role of the wealth index, which showed no significant correlation with the time to drop out of CBHI. The results of the existing literature showed that the socio-economic status of households was significantly linked to membership renewal decisions, regardless of whether it was measured in terms of asset category, expenditure, or income. Households with higher economic status were more likely to renew their policy compared to those with lower economic status [13, 14, 17, 22, 26, 34]. This difference might be linked to the study population and premium affordability. Our study excludes indigents that are fee waiver beneficiaries, which otherwise could not adhere to the scheme membership if they were made to pay the premium. A prior study in Ethiopia stated that the bulk of households were able to afford the premium [18], which might be in part due to deliberate government efforts to provide a fee waiver for the poorest segments of the population. Another study documented that level of premium affordability was not significantly associated with dropout decisions [19]. A household’s ability to afford the scheme is a function of its economic status. As long as premium affordability is not the concern of the population, the economic status of the household might not have a role in influencing continued membership adherence. In line with our finding, recent work in Ethiopia reported that there was no significant link between the wealth index and policy renewal [19]. This finding implies the role of providing fee waivers for indigents in assuring the equity goal of universal health coverage. To sum up, the findings of this study on some of the predictors of time to drop out are consistent with the literature, whereas for some other predictors it contradicts with studies conducted in other countries. It could be understood that the study setting, including the scheme design (premium affordability, level of premium contribution per household size, and fee waiver policy) and the study population, which includes only paying scheme members, had an important bearing on the observed discrepancy.

Despite the study provides useful insights into CBHI and other voluntary insurance schemes, it is not without limitations. The study might be prone to recall bias in the measurement of the perceived quality of health care. Some respondents who had no recent visit to public health facilities, might not be as critical as those who had a recent visit experience. Second, the items set to measure value towards solidarity might also be subjected to social desirability bias, for which most respondents might rate the items higher than their true feelings which could narrow the variation. Third, since the factors and the outcome of interest were measured at the same point in time, there exists the possibility of reverse causality.

## Conclusions

The presence of chronic illness in the family and household size were significant and positive predictors of the membership duration in CBHI, pointing out the existence of adverse selection. In the latter one, it appears the problem is related to the design of the premium which attracts larger households to the scheme. This indicates the need to reconsider the premium level in line with household size. Despite CBHI is enhancing health care access to its members particularly for high-risk individuals living with chronic conditions, it lacks the ability to protect them from financial hardships at the time of receiving health care. This implies that the scheme could not be able to generate adequate funds to satisfy the health care needs of its members. History of hospitalization under the scheme was a positive predictor of the time to drop out of CBHI, which implies that those who enjoyed the benefit of health insurance prefer to maintain their subscription. The findings of this study have implications for addressing issues related to health care quality. Unless scheme members are receiving good quality health care under the scheme, they might lose trust and develop a negative attitude towards the scheme.

## Data Availability

The datasets used and/or analyzed during the current study are available from the corresponding author on reasonable request.

## Abbreviations

AFT: Accelerated Failure Time
AIC: Akaike’s Information Criterion
BIC: Bayesian information criterion
CBHI: Community-Based Health Insurance
ODK: Open Data Kit
SD: Standard Deviation
